# Validation of the German version of the Family Reported Outcome Measure (FROM-16) to assess the impact of disease on the partner or family member

**DOI:** 10.1186/s12955-021-01738-4

**Published:** 2021-03-24

**Authors:** Susanne A. Elsner, Sam S. Salek, Andrew Y. Finlay, Anna Hagemeier, Catherine J. Bottomley, Alexander Katalinic, Annika Waldmann

**Affiliations:** 1 Institute for Social Medicine and Epidemiology, University of Luebeck, Ratzeburger Allee 160, 23562 Luebeck, Germany; 2 School of Life and Medical Sciences, University of Hertfordshire, Hatfield, AL10 9AB UK; 3 Outcomes Research, Institute for Medicines Development, Cardiff, CF23 6NP UK; 4 Division of Infection and Immunity, School of Medicine, Cardiff University, Cardiff, CF14 4XN UK; 5 Vitaccess, Centre for Innovation, New Road, Oxford, OX1 1BY UK; 6 Cancer Registry Schleswig-Holstein, University of Luebeck, Luebeck, Germany

**Keywords:** Quality of life, FROM-16, Partner, Family members, Questionnaire translation, WHOQOL-BREF (GHS), Informal caregiving

## Abstract

**Background:**

The Family Reported Outcome Measure (FROM-16) assesses the impact of a patient’s chronic illness on the quality of life (QoL) of the patient’s partner or family members. The aim of the study was to translate, explore the structure of and validate the FROM-16.

**Methods:**

The questionnaire was translated from English into German (forward, backward, four independent translators). Six interviews with family members were conducted to confirm the questionnaire for linguistic, conceptual, semantic and experiential equivalence and its practicability. The final German translation was tested for internal consistency, reproducibility and test validity. Criterion validity was tested by correlating the scores of the FROM-16 and the Global Health Scale (GHS). Principal component analysis, factor analysis, and confirmatory factor analysis was used to assess the questionnaire’s structure and its domains. Reliability and reproducibility were tested computing the intraclass correlation coefficient (ICC) using one sample t-test for testing the hypothesis that the difference between the scores was not different from zero.

**Results:**

Overall, 83 family members (61% female, median age: 61 years) completed the questionnaire at two different times (mean interval: 22 days). Internal consistency was good for the FROM-16 scores (Cronbach’s α for total score = 0.86). In those with stable GHS, the ICC for the total score was 0.87 and the difference was not different from zero (*p* = 0.262) indicating reproducible results. A bi-factor model with a general factor including all items, and two sub-factors comprising the items from the original 2-factor construct had the best fit.

**Conclusions:**

The German FROM-16 has good reliability, test validity and practicability. It can be considered as an appropriate and generic tool to measure QoL of a patient’s partner or family member. Due to the presence of several cross-loadings we do not recommend the reporting of the scores of the two domains proposed for the original version of FROM-16 when using the German version. Thus, in reporting the results emphasis should be put on the total score.

*Trial registration*: Retrospectively registered: DRKS00021070.

**Supplementary Information:**

The online version contains supplementary material available at 10.1186/s12955-021-01738-4.

## Background

Among the 3.41 million people in need of care in Germany in 2017, about 1.76 million were cared for at home exclusively by partners or relatives [1]—so called informal caregivers. Almost two thirds of the caregivers (64.9%) were female and one third (35.1%) male. The share of informal caregivers is highest in the 55–69 age group [2]. The majority of those in need of care (63%) were women. 81% were 65 years or older and 35% were 85 years or older [1]. The average duration of care for all age groups is 6.7 years, and that reduces to 4.4 years for people who become in need of care at the age of 60 or older.

These finding suggests that the majority of the people in the need of care suffer from chronic diseases. Chronic diseases are long-lasting diseases that cannot be fully cured and lead to a continuous or recurrent increase in the use of health care services. Aside from the need of continuous medical treatment and supervision, a huge proportion of chronically ill people are in need of care i.e. support regarding daily activities and self-care. It should be noted that a uniform definition of the term “chronic disease” does not exist [3]. Examples of chronic diseases include cardiovascular diseases such as coronary heart disease and strokes, diabetes, cancer and chronic respiratory diseases. In Germany, these diseases account for three quarters of deaths and around one quarter of medical expenses [1].

Compared to the wealth of evidence regarding the quality of life (QoL) of patients, only a very small number of studies have examined the QoL of partners or family members acting as informal caregivers. As it has been pointed out by Golics et al. [4, 5] and others [6, 7] a significant number of informal caregivers have complex, multidimensional and/or significant unmet needs and experience detriments in the quality of life and health status. Such limitations have been described, for example, in terms of physical (back pain) and emotional and mental health, such as feelings of anxiety, depression, helplessness, fatigue, or the feeling to be obliged to give care [2, 5, 8, 9].

Further, a wide variety of aspects of family members’ lives can be affected. In particular, financial well-being can be affected by the additional costs for re-modeling the house to accommodate a disabled family member, for transport or extra medical costs. Family relationships may also suffer from the particular situation, as the roles and tasks of family members may change due to the circumstances. This can result in a lack of understanding of each other’s feelings and dependency relationships. Education and work may also be affected, as it may be necessary to reduce working hours or leave work either temporarily or completely in order to have time to care for the relative. In addition, the caregiver may be unable to concentrate because of worries, or may spend time at work talking to relatives on the phone. Finally, leisure and social activities can also be affected due to the responsibility. Some caregivers may experience separation from their friends because of the feeling that their own live is so different from that of their friends [4–6, 10–13].

There are relatively few standardized instruments that focus on the QoL of informal caregivers or family members. One of these is the Family Reported Outcome Measure (FROM-16). It was developed by Golics et al., based on information from partners and family members of patients from 26 different medical specialties. It has been tested for feasibility, practicability, test validity and reliability [14]. In comparison to existing questionnaires, the FROM-16 is characterized by its briefness (16 items) and a clear focus on family issues. The difference between the FROM-16 and other QoL instruments is that FROM-16 specifically asks about the impact on QoL of the caregivers. It therefore is designed to filter out other QoL issues which are not related to having a family member with a health condition. FROM-16 is currently the only generic QoL instrument to target and measure the secondary burden of disease on family members.

Because there has been no standardized, validated German questionnaire assessing the QoL of informal caregivers (most of them being family members), we aimed to translate FROM-16 from English into German and validate it in a group of caregiving partners or family members of patients with different diseases. Our hypothesis is that the German version of FROM-16 would have the same psychometric characteristics as the original English version, including its two-factor structure, as well as its convergent validity with closely related measures.

## Materials and methods

### FROM-16

FROM-16 [14] includes 16 items grouped into two dimensions. The first assesses the subject’s emotional state (items 1 to 6) and the second focusses on the impact on everyday life and relationships (items 7 to 16). Each item is answered using a three-point Likert scale (English: *not at all, a little, a lot;* German*: gar nicht, ein wenig, sehr*) and scored with 0, 1 or 2 points, respectively. The total score is the unweighted sum of all responses and ranges from 0 to 32 (i.e. 0 to 12 for the “Emotional” dimension; and 0 to 20 for the “Personal and Social Life” dimension). A high score indicates a greater negative impact on QoL.

### Translation process

Four independent translations of the original questionnaire were obtained. Two of them were made by professional English language translators. The remaining two translations were made by the authors AW (epidemiologist) and SE (social scientist), both experienced in health care research. All translators were native German speakers. The different translations were discussed and revised by the translation team during one joint meeting. Finally, one German version was developed. This questionnaire was translated backwards independently by two professional English translators, one a native English speaker, who had not previously been involved. Both back-translated versions were reviewed by a member of the copyright holders’ team (Dr. FM Ali). The differences between the original and the translated versions were discussed and a pre-final version of the German questionnaire was defined.

### Recruitment of participants

We recruited partners and family members with a minimum age of 18 years who gave informal care to a patient with a chronic disease. Due to the lack of a unique definition of the term “chronic disease” and as we wanted to sample a heterogeneous group of patients, we did not specify in- and exclusion criteria with regard to specific diseases. In order to select participants who provided informal care to patients from different clinical specialties, we used multiple recruitment methods: advertisements in newspapers and local magazines, the newsletter and the webpage of the University of Luebeck, and researcher visits in different clinical consulting hours, outpatient care services and day-care hospitals. We got further support from heads of self-help groups for caregiving family members, who provided study information to potential participants. We recruited 96 subjects of whom six took part in the cognitive debriefing interviews and 90 in the validation study.

### Procedure for the cognitive debriefing interviews—face validity

The pre-final German version of FROM-16 was first tested by carrying out cognitive debriefings of adult relatives of chronically or seriously ill patients. The patients’ diseases included stroke, brain tumour, schizophrenia, dementia or heart failure. This was to test the linguistic, conceptual, semantic and experiential equivalence and the practicability of the translated questionnaire. Since five to 15 participants are deemed to be appropriate for cognitive debriefing [15], we recruited six interview partners (three female and three male (mean age: 59 years, range: 29–86).

The participants were asked to express their opinion about each item, including any problems that they perceived, in order to improve the quality of the FROM-16 translation. All interviews were recorded. Participants received an expense allowance of 20 Euros.

Different techniques of questioning such as comprehension probing, category selection probing, confidence rating, paraphrasing and thinking aloud techniques were used [15]. At the end of the interviews, participants were asked about their overall impression of the questionnaire, such as “was it easy or difficult to complete?” or “was something important missing?”.

Following this process, the title was slightly changed (from “Erhebung familienberichteter Daten zur Lebensqualität” to “Familienberichtete Daten zur Lebensqualität”) and one question was reworded. All partners interviewed stated that the FROM-16 was easy to understand and quick to complete. The most prevalent comments referred to three items. One unfavorable comment was made concerning the term “everyday travel”. We found no German equivalent and translated it with “mobility” (German: “*ist meine tägliche Mobilität betroffen*”). Nearly all participants interpreted “mobility” with “physically exhausting”, so that we had to clarify the target of the question with an additional explanation (“*Verrichtung der täglichen Wege*”). Another frequent comment referred to the item “*Caring for my family member is difficult*” (Deutsch: “*Es ist schwierig, für mein Familienmitglied zu sorgen*”). Some participants wondered if they should interpret it in a physical, psychological or financial manner. The last issue concerned item twelve (“*My sex life is affected*”). Some people mentioned that intercourse—what most of them understood by “sex life”—was not so important but what counted was tenderness and caresses. After discussion with the copyright holders it was decided not to change the two latter questions. An open interpretation was considered useful because everyone experiences different constraints and a generic use of FROM-16 was intended.

### Procedure for test–retest-reliability/reproducibility study

90 adult participants were recruited for the reproducibility study. All were caregiving relatives of patients with various diseases (Table 1). 83 of them repeated the German FROM-16. Based on a difference of 0.15 between minimal accepted (ICC = 0.7 [16]) and expected reliability (ICC = 0.85), alpha of 0.05, beta of 0.2, and a dropout of 10%, a sample size of 59 would have been sufficient [17].

**Table 1:** Demographics of the family members/informal caregivers

Family members	Test/t_1_ [n = 90]	Retest/t_2_ [n = 83]
Gender		
Male	34 (37.8%)	32 (38.6%)
Female	56 (62.2%)	51 (61.4%)
Age (years)		
Mean (± SD)	59.6 (± 14.4)	59.6 (± 14.7)
Median (interquartile range)	61 (51–71.5)	62 (51–72.3)
Relationship to patient		
Partner	54 (60.0%)	50 (60.2%)
Parent	20 (22.2%)	18 (21.7%)
Child	10 (11.1%)	10 (12.0%)
Siblings	1 (1.1%)	1 (1.2%)
Other	4 (4.4%)	3 (3.6%)
Missing information	1 (1.1%)	1 (1.2%)
Living in one household with the “person to be taken care of”		
Yes	72 (80.0%)	64 (77.1%)
No	17 (18.9%)	19 (22.9%)
Missing information	1 (1.1%)	0
Civil status		
Single/living alone	8 (8.9%)	8 (9.6%)
Married	67 (74.4%)	62 (74.7%)
In a permanent relationship	6 (6.7%)	6 (7.2%)
Separated	1 (1.1%)	1 (1.2%)
Divorced	7 (7.8%)	4 (4.8%)
Widowed	1 (1.1%)	2 (2.4%)
Level of education		
Education (≤ 10 years)	48 (53.3%)	45 (54.2%)
Education (> 10 years)	22 (24.4%)	21 (25.3%)
Higher Education (University, PHD)	20 (22.2%)	17 (20.5%)
Condition of the informal caregiver^a^		
Fit/healthy	52 (57.8%)	48 (57.8%)
Diseased	37 (41.1%)	35 (42.2%)
Missing information	1 (1.1%)	0
Diagnosis of the patients whom informal caregivers take care of (multiple ticks were allowed)		
Cardiovascular	9 (10.0%)	
Old age frailty	2 (2.2%)	
Chronic pain	2 (2.2%)	
Endocrinology	8 (8.9%)	
Mental health	25 (27.8%)	
Neurology	45 (50.0%)	
Oncology	18 (20.0%)	
Respiratory	5 (5.6%)	
Orthopedics	4 (4.4%)	
Nephrology	2 (2.2%)	
Genetics	4 (4.4%)	

^a^The health status of the family member was assessed with one closed question (“Are you currently ill?” [Answer options: yes/no]) and with one open question (“If yes, which illness do you have?”)

The first questionnaire (t_1_) was either handed out to study participants in person (90% of the study participants) or emailed together with the study information, an informed consent form and a stamped envelope. After receiving the first questionnaire, we sent the second questionnaire (t_2_) usually within 10 to 14 days. Terwee et al. (2007) recommend a time interval of one to two weeks between repeating the questionnaire—long enough to prevent recall, but short enough to ensure small but clinically important change [16].

If there was no response within two weeks, participants were sent up to two reminders. Participants received 10 Euros when both questionnaires were completed and sent back.

### Study questionnaire for the reliability and validation study

The questionnaire included the FROM-16 [14] and the German WHOQOL-BREF, with the integrated Global Health Score (GHS). It consists of 26 items grouped into four domains (score range after transformation 0 (worst) to 100 (best value). The psychometric properties have been tested with cross-sectional data of adults from 23 countries. Regarding internal consistency, Cronbach’s α were acceptable (i.e. > 0.7) in the domains of physical health (0.82), psychological (0.81), environment (0.80), but marginal for social relationships (0.68). Further, the instrument proved to have discriminant validity when comparing “ill” and “well” subsamples [18–20].

We asked the study participants to assess their and the family members’ GHS-score in proxy (0 to 10 on a visual analogue scale, with 0 = worst possible health and 10 = perfect health). In order to check if possible changes were associated with substantial differences regarding the patients’ health between t_1_ and t_2_, respondents were asked in the retest questionnaire (t_2_) if the family members’ health status had changed compared to t_1_ (answer options: *equal, deterioration* or *improvement*).

The health status of the family member was assessed with one closed (“Are you currently ill?” [Answer options: yes/no]) and with one open question (“If yes, which illness do you have?”).

### Statistical analysis

For the descriptive analysis common measures of location and dispersion and absolute and relative frequencies were calculated. In analogy to the psychometric testing process of the original FROM-16 [14], measures of internal consistency, reproducibility, criterion and construct validity were calculated.

Since exploratory factor analysis revealed a two-factor structure for the original, the English FROM-16 [14], we first aimed to confirm this structure for the German FROM-16. However, none of our approaches (e. g. Confirmatory Factor Analysis (CFA), Principal Component Analysis (PCA); *R packages psych and lavaan*) was able to confirm the two-factor structure. Moreover, it became obvious that a one-factor structure would best fit the structure. Therefore we run a bi-factor model and an unrestricted parallel analysis based on minimum rank factor analysis [21] (PA; dispersion matrix: polychoric correlations [22, 23] using *R package lavaan* [24] and the software FACTOR [25–27]. The indicator variables followed a multivariate normal distribution. Maximum likelihood estimation was used, with full information maximum likelihood (FIML) for the missing data and standardized latent factors, allowing free estimation of all factor loadings. To assess model fit, the root mean square error of approximation (RMSEA; recommended to be < 0.06), the standardized root mean square residual (SRMR; recommended to be < 0.08) were calculated, as well as the comparative fit index (CFI) and Tucker-Lewis Index (TLI). For the latter, values in the range of 0.90 to 0.95 may indicate an acceptable model fit [28]. In addition to RMSEA and PFI, for PA the very simple structure (VSS) criterion, Velicer’s MAP and Schwarz’s Bayesian Information Criterion (BIC) values were used to assess the model fit. The number of factors which maximize the VSS criterion is taken as being the optimal number of factors to extract, while the lowest values for Velicer’s MAP and BIC indicate the model with the best fit.

Internal consistency was assessed by Cronbach’s α [29], and to be judged as good, α should be at least 0.7 [16].

Test–retest reliability was assessed by investigating whether the FROM-16 scores remained unchanged when administered to family members with a stable health status on two occasions. Health status was considered to be stable if the GHS had not changed by more than 1 point between test and retest. In total, 64 respondents had a stable GHS score and were included in the reproducibility analysis using the intra class correlation coefficient (ICC) based on single measurements with the following specifications: model “2-way mixed model” and type “absolute agreement” [30]. To be judged as good, the ICC should be at least 0.7 [16]. In addition, the difference between t_1_ and t_2_ scores was calculated. Using the one sample t-test, we tested whether this difference was statistically significant from zero.

In order to test the criterion validity the total score and the domains’ scores were correlated to the GHS score. As the data were skewed on an ordinal scale, Spearman r was computed. To be judged as good, r should be at least 0.7 [16].

Furthermore, we assessed differential item functioning, which describes whether mean FROM-16 scores differed between known subgroups, such as gender- and age-specific subgroups and the disease of the patients who were cared for by the informal caregivers. For this we used Student’s t-test for unpaired data and ANOVA (age categorized according to quartiles: ≤ 51, 52–61, 62–71, and ≥ 72 years), respectively. Linear regression was used to assess the independent effect of gender (using the original metric data) of the informal caregivers and disease of the patient.

Data were analyzed using IBM SPSS Statistics (Version 22) and R 3.5.0 [31]. The R packages *psych* [32] and *lavaan* [24] were used to conduct the PCA, CFA and PA. Further, the software FACTOR [25–27] was used to confirm the results of PA achieved with R. DIF analysis was performed with IRTPro v4.2.

### Readability

The Flesch-Reading-Ease Score (FRE-score; Flesch-Index) measures the complexity of sentences. The score ranges from 0 to 100. High values indicate a level of “easy reading” and low values indicate more “complex sentence structures”. As the English and the German language differ in sentence structure, sentence and word length, different formulae were used to compute the FRE-score (Eq. 1).

**Equation 1:** Formulae for computing the Flesch-Reading-Ease-Score for the English [33] and the German FROM-16 [34]

ENGLISH:	
FRE = 206.835 − 84.6 * WL − 1.015 * SL	
GERMAN:	
FRE = 180 − SL − WL * 58.5	
WL = average word length in syllables	
(= number of syllables divided by number of words)	
SL = average sentence length	
(= number of words divided by number of sentences)	

## Results

### Demographics

Of 90 initially recruited participants, 83 completed the questionnaires at t_1_ and t_2_. The median age of the participants was 61 years (interquartile range = 51–71.5) and 62% were female. The relationships of the subjects to the patients were most commonly “partner” followed by “parent” or “child”. Patient’s most common diseases were neurological or psychiatric disorders followed by oncological diseases (Table 1).

### Validity

#### Factorial validity/internal consistency

Table 2 displays factor loadings derived with exploratory factor analysis (EFA) for the English and for the German version of FROM-16 and results for the equivalent 2-factor confirmatory factor analysis (CFA; see also Table 3). The number of cross-loadings was high in EFA.

**Table 2:** Structure matrix of the German FROM-16 showing the loading of each item onto the two factors

Item number	Item description	Exploratory Factor Analysis—English questionnaire (Golics et al. 2014)		Exploratory Factor Analysis—German questionnaire		Equivalent 2-factor Confirmatory Factor Analysis—German questionnaire
		Emotional	Personal and social life	Emotional	Personal and social life	Beta (standardized parameter estimates)
Emotional domain						
1	I feel worried	0.696	–	0.670	0.402	0.524
2	I feel angry	0.766	0.531	0.606	0.314	0.590
3	I feel sad	0.861	0.434	0.753	0.330	0.650
4	I feel frustrated	0.814	0.508	0.662	0.345	0.611
5	It is difficult to find someone to talk to	0.613	0.519	0.512	-0.036	0.268
6	Caring for my family member is difficult	0.461	0.503	0.435	0.579	0.637
Personal and social life domain						
7	It is hard to find time for myself	0.466	0.697	0.351	0.782	0.632
8	My every day travel is affected	0.575	0.743	0.230	0.717	0.552
9	My eating habits are affected	0.446	0.761	0.306	0.652	0.583
10	My family activities are affected	–	0.761	0.582	0.503	0.643
11	I experience problems with going on holiday	–	0.700	0.426	0.497	0.524
12	My sex life is affected	0.530	0.687	0.313	0.351	0.252
13	My work or study is affected	0.437	0.769	-0.067	0.715	0.463
14	My relationships with other family members are affected	0.517	0.723	0.463	0.524	0.554
15	My family expenses are increased	–	0.768	0.312	0.703	0.526
16	My sleep is affected	0.540	0.640	0.544	0.549	0.585

Extraction method: Principal component analysis. Rotation method: Oblimin with Kaiser Normalization

**Table 3:** Fit details of 1-, 2- and 3-factor CFA models and a bi-factor model

Model	Degrees of freedom	p-value (x^2^)	CFI	TLI	RMSEA	Lower	Upper	SRMR	AIC	BIC
1-Factor (1 to 16)	104	< 0.001	0.766	0.730	0.095	0.074	0.017	0.086	3800.8	3920.7
2-Factor (factor 1 = items 1 to 6; factor 2 = items 7 to 16)	103	< 0.001	0.788	0.753	0.092	0.069	0.113	0.085	3793.7	3916.2
3-Factor (factor 1 = items 1 and 3; factor 2 = items 2, 4, 5, 6; factor 3 = items 7 to16)	101	< 0.001	0.794	0.755	0.091	0.067	0.113	0.082	3793.1	3930.6
Bi-factor (general = all; factor 1 = items 1 to 6; factor 2 = items 7 to 16)	87	0.002	0.872	0.823	0.082	0.047	0.100	0.074	3114.7	3227.5

In principal component analysis (PCA) when only the first two components were considered, 43.7% of the variance was explained. However, the fit statistics showed only poor fits. Models with more than two factors gave better fit statistics, but did not support the psychosocial construct of the FROM-16 conceptual framework with two domains. Further investigation into 1-, 2- and bi-factor solutions was therefore needed and consequently 1-, 2- and 3-factor CFA models and bi-factor models (*R lavaan omega and omega hierarchy modelling*) were explored. The details are shown in Table 3.

**Fig. 1:**
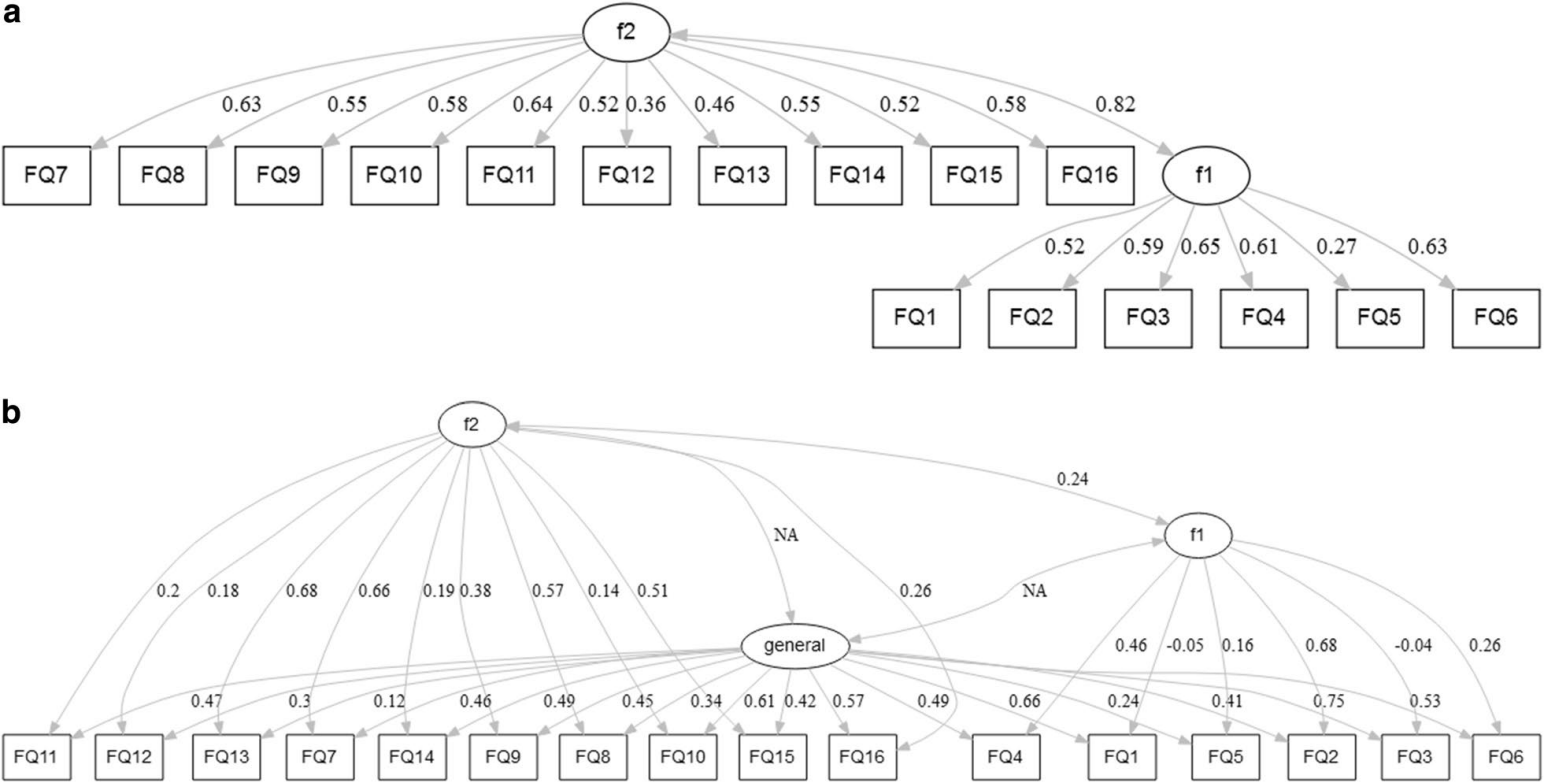
Diagrams for 2-factor and bi-factor models

The bi-factor model with the best fit (*shown*) revealed high loadings onto a single factor for nearly all of the items (*p* < 0.05; loadings > 0.3 except items 5 and 13), with items 2, 4 and 6 loading onto the first factor, and items 7, 8, 9, 13, 15 and 16 loading onto two sub-factors (*p* < 0.05). The details of this model are shown in Fig. 1.

**Fig. 2:**
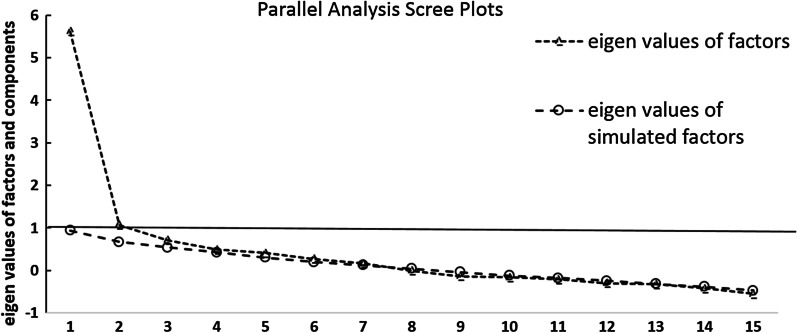
Parallel Analysis Scree Plots for baseline measurement (t_1_)

The unrestricted parallel analysis using a polychoric correlation matrix (the Kaiser–Meyer–Olkin test for adequacy of using this matrix gave a value of 0.851) confirmed the one factor solution, with BIC of 298.344, RMSEA of 0.086 and CFI of 0.955 for the one factor solution; VSS criterion reached a maximum (0.71) and Velicer’s MAP (0.02) achieved a minimum for this solution. The parallel analysis scree plot is shown in Fig. 2.

The internal consistency was good for the total score (Cronbach’s α = 0.86 (t_1_); Table 4).

**Table 4:** Descriptive statistics for FROM-16-scores and GHS-scores baseline (t_1_) and retest (t_2_) and measures of reliability and criterion validity

	t_1_ FROM-16 Total Score	t_1_ caregivers General Health Score (GHS)	t_2_ FROM-16 Total Score	t_2_ caregivers General Health Score (GHS)
Valid answers	74	90	67	83
Mean (SD)	16.8 (6.6)	6.1 (1.9)	16.1 (7.2)	6.1 (2.0)
Median (interquartile range)	17 (12–21.3)	6 (5–8)	16 (11–21)	6 (5–8)
Min–Max	0–31	2–10	0–31	0–10
Floor	1	–	2	1
Ceiling	–	2	–	2
Internal consistency:				
Cronbach’s α	α = 0.859		α = 0.894	
Reproducibility (t1 and t2):				
ICC (95%CI)	0.87 (0.79–0.92)			
Criterion validity:				
Correlation of Total score with…		r = − 0.35 *p* = 0.002		r = − 0.48 *p* < 0.001
Correlation of Emotional score with…		r = − 0.32 *p* = 0.003		r = − 0.39 *p* = 0.001
Correlation of Personal and Social Life score with…		r = − 0.33 *p* = 0.003		r = − 0.48 *p* < 0.001

#### Minimal analysis partial test

The results of the MAP analysis (Additional file 1: Table S1) strengthens the one-factor argument, with an average square d partial correlation coefficient of 0.07 and average 4th power partial correlation coefficient of 0.015 compared to values < 0.5 and < 0.005, respectively, for solutions with more factors.

#### Differential item functioning by Gender

Females scored significantly higher values for the total score and the domains scores than males (total, females: mean = 18.3, SD = 6.2 vs. males: mean = 14.5, SD = 6.6, *p* = 0.015; “Emotional” domain, females: mean = 7.2, SD = 2.4 vs. males: mean = 5.8, SD = 2.8, *p* = 0.015; “Personal and Social Life” domain, females: mean = 11.0, SD = 4.6 vs. males: mean = 8.6, SD = 4.3, *p* = 0.021). When adjusting for gender, no association between age and the FROM-16 scores using either bivariate or multivariable analyses was observed.

From frequencies of item distributions, responses were well distributed across the three levels. S-X2 Item level Diagnostic statistics showed good IRT fit for the reference group (female, N = 56) with only item 10 *p* < 0.05, but poorer fit for focal group (male, N = 34) with 8 poorly fitting items *p* < 0.05. Furthermore, data showed no local dependency with All Maximum Standardized LD X2 Statistics for all reference and focal group items < 3.6. Statistics based on one- and two-way marginal tables showed good fit with RMSEA = 0.06.

Test of all items with all items anchored showed no DIF from DIF statistics for the graded items (Model 1, 2) (Table 5). Testing candidate items one at a time and estimating group difference with all other items anchored showed no DIF from DIF statistics. Test characteristic curves for the expected values of two group showed little difference, particularly between theta = 2 (item with moderate level of ease) and theta = -2 (items with moderate level of difficulty). Item test characteristic curves showed good match between the two DIF groups and standard errors were < 0.5 (good) over most of the theta range for both groups.

**Table 5:** Differential Item Functioning (DIF) by gender

Test all items, anchor all items. DIF Statistics for Graded Items											
Item numbers and description in:	Group 1	Group 2	Total *X*^2^	*d.f*	*p*	*X*^2^_a_	*d.f*	*p*	*X*^2^_c|a_	*d.f*	*p*
1. I feel worried	1	1	1.3	3	0.7370	0.9	1	0.3349	0.3	2	0.8455
2. I feel angry	2	2	2.8	3	0.4297	0.4	1	0.5332	2.4	2	0.3054
3. I feel sad	3	3	4.5	3	0.2100	1.5	1	0.2230	3	2	0.2187
4. I feel frustrated	4	4	1.1	3	0.7806	0.1	1	0.7755	1	2	0.6054
5. It is difficult to find someone to talk to	5	5	1.1	3	0.7715	0.3	1	0.5634	0.8	2	0.6742
6. Caring for my family member is difficult	6	6	0.7	3	0.8768	0.7	1	0.4138	0	2	0.9920
7. It is hard to find time for myself	7	7	1.1	3	0.7810	0.1	1	0.8194	1	2	0.5971
8. My every day travel is affected	8	8	1.7	3	0.6403	0.4	1	0.5501	1.3	2	0.5151
9. My eating habits are affected	9	9	4.1	3	0.2515	0.1	1	0.8035	4	2	0.1322
10. My family activities are affected	10	10	2	3	0.5747	1.3	1	0.2592	0.7	2	0.6998
11. I experience problems with going on holiday	11	11	2.5	3	0.4713	1.6	1	0.2082	0.9	2	0.6264
12. My sex life is affected	12	12	4.2	3	0.2435	0	1	0.8998	4.2	2	0.1242
13. My work or study is affected	13	13	4.4	3	0.2195	2.5	1	0.1177	2	2	0.3736
14. My relationships with other family members are affected	14	14	0.3	2	0.8460	0.1	1	0.7198	0.2	1	0.6503
15. My family expenses are increased	15	15	1.5	3	0.6919	0.5	1	0.4871	1	2	0.6143
16. My sleep is affected	16	16	2.7	3	0.4495	1.5	1	0.2142	1.1	2	0.5768

*p* values are for the Wald χ^2^-statistic that tests the difference between reference and focal group item parameters

χ^2^_a_ for slope (discrimination)

χ^2^_c/a_ for location

Significant DIF = *p* < 0.05

#### Criterion validity

A moderate inverse correlation was found for the FROM-16 total score and the GHS of the participants. Similar levels of correlation were observed when the participants FROM-16 total score of was correlated to the GHS proxy-reports for the patients (Total score: r = -0.50, *p* < 0.001; Table 4).

### Reliability

#### Test–retest-reliability/reproducibility

Valid information on the test–retest interval is available for 67 study participants. Twenty-nine (43%) participants did not return the retest questionnaire (t_2_) within 14 days. Twenty (31% of 29 participants) responded to the first reminder, and two participants answered after a second reminder. The remaining seven participants did not respond. The mean test–retest interval was 22 days (SD = 9).

Of all 83 participants, 64 (71%) completed the FROM-16 at t_1_ and t_2_ and reported no change in their global health status (difference on the GHS between t_1_ and t_2_ not more than one point). The ICC for the total score was based on evaluable responses of 46 persons allowing the calculation of the scale score for t_1_ and t_2_. The mean difference of the values between t_1_ and t_2_ was 0.54 and was not statistically significant from zero (*p* = 0.262). The ICC was 0.87 (95%CI = 0.79–0.92) and thus also suggesting reproducible results in participants with stable health status (Table 4).

#### Measurement properties

The German FROM-16 total score at baseline (t_1_) ranged from 0–31 with a median value of 17 (interquartile range = 12–21.3). A ceiling effect was not apparent on the total score or on the “Emotional” score, while three participants scored the highest possible value on the “Personal and Social Life” score. Only one person scored zero on the total score, while two persons each scored zero on the “Emotional” and the “Personal and Social Life” score indicating a minimal floor effect (Table 4). Items with the highest number of missing values were those expressing extreme feelings (item “I am angry” t_1_: 6 missing values, t_2_: 5 missing; item “I am frustrated, t_1_: 2 missing, t_2_: 5 missing) and the item addressing work and study relations (t_1_: 10 missing, t_2_: 5 missing). The age range of the participants with the work item missing was 66 to 84 years.

#### Readability

The mean length of the 16 items in the German version of FROM-16 was 5.9 words (range = 3–14) and 5.6 words (range = 3–12) for the English version. The FRE-score of the German version was 54.8 and thereby lower than the FRE-score of the original English version (61.8). This indicates a slightly more complex structure (and thus poorer readability) for the German compared to the English version.

## Discussion

The voluntary support and caregiving to a family member can be seen as a positive and important contribution to a functioning partnership or to family life. However, informal caregiving can also cause severe health problems and psychological impairment. As there is sparse information, a practical instrument to assess the impact and possible burden of informal caregiving on partners and relatives is needed. Due to a lack of a validated questionnaire in the German language, we translated the FROM-16 from English into German and tested the German FROM-16 for linguistic, conceptual, semantic and experiential equivalence. The practicability, internal consistency, reproducibility and test validity was also tested. The validation process was performed according to the psychometric testing procedure of the original instrument [14] and further extended. However, for the purpose of this manuscript we have chosen not to formally assess the measurement equivalence of the English and the German scale. Therefore, it is currently unknown whether the German and English scales possess the same metric and intercept. Further research to assess differential item functioning is warranted.

Nearly all interviewees considered that the German FROM-16 was easy to understand and quick to fill in. Although the items 6 and 12 were perceived as somewhat unclear by some interviewees, we did not specify the meanings these items (“*Caring for my family member is difficult*” and “*My sex life is affected*”), because of the intended generic nature and use represented by the original scale. All participants of the test–retest reliability study completed the German FROM-16 correctly and there were no signs of the existence of systematic errors.

The age structure and the ratio of females to males in our sample correspond well to the average characteristics of caregivers presented by the Robert Koch-Institute, Germany [2]. With regard to these characteristics, we can therefore assume that our sample is well representative.

The target group of FROM-16 are adults. However, especially the item relating to work and studies might be difficult to answer for older participants and for those who are not working. Missing values on this item can be expected when the questionnaire is completed by retired or unemployed persons.

The original English version of FROM-16 has two domains. The separation precision between the two domains in the German version was not distinct regarding the majority of items. Thus, it should be noted, that the original English version also had weaknesses in terms of the two domain structure, which was demonstrated by several cross loadings. The two-domain structure proposed for the original English version of FROM-16 [14] and the recently published Thai FROM-16 [35] could therefore not be confirmed for the German version. The best fit to the data was achieved by using a bi-factor model with a general factor that included all items, and two sub-factors that comprised the items from the original 2-factor construct. The bi-factor model is a form of confirmatory factor analysis applied in a generalised manner to the case of discrete item-response data [36]. It requires that each item loads on a primary dimension of interest and no more than one secondary dimension such as positively or negatively worded items (i.e. methodologic factor) or content domain from which the items are sampled (e.g. component dimensions underlying the overall quality of life). The bi-factor model has the potential of providing numerous advantages over an unrestricted exploratory item factor analysis model [37]. Further unrestricted parallel analysis, VSS and MAP suggest the one-factor solution based on fit to the data, too. There is significant cross loading of some items in 2 and 3-factor models, and fit statistics are very similar to the 1-factor solution. However, fit statistics should significantly improve with increasing number factors if they truly exist, which was not the case for our data. In view of our findings we propose to use the total score when reporting the results of the German FROM-16 rather than the scores of the originally proposed two subscales.

The psychometric validation of the German FROM-16 total score revealed somewhat lower results, but in strength, direction and interpretation quite similar results to the findings of the validation study of the original FROM-16. Internal consistency was demonstrated by a relatively high Cronbach´s α for the total score. It was slightly lower than in the original reliability study (total score α = 0.91), which might be due to the more heterogeneous study sample and the more restricted range of patients’ diseases in the German study [14]. Nevertheless, we do not consider this small difference to be relevant and with Cronbach’s α > 0.7 the internal consistency of the German FROM-16 can be rated as excellent.

The inverse correlation between the FROM-16 and the GHS scores suggests that the self-assessed health status of the family member affects the ratings of their QoL. This inverse correlation was also seen when they assessed the GHS score of the patient as a proxy. The lower that the family member rated the patients’ QoL, the higher was the impact on QoL ratings. Thus, both the health of the patient and the health of the family member are correlated to the impact of the patient’s illness on the family member’s QoL.

Test–retest reliability was high. The ICCs were lower compared to the original version (total score ICC = 0.93), which might be due to differences between the two study populations. Nevertheless, with an ICC value > 0.7 in the present study and with the difference between t1 and t2 scores not being statistically significant from zero, the reliability of the German FROM-16 can be rated as good.

The use of FROM-16 is likely to reveal aspects of burden experienced by family members that were previously hidden. Information from FROM-16 could be used to target appropriate support to affected family members. There may be circumstances revealed by FROM-16 where management of patients may need to be adjusted to address these secondary issues. In addition, FROM-16 data may be used as a secondary outcome measure in the evaluation of new treatment interventions.

### Strengths and limitations

This study’s strengths are that it included a thorough translation procedure involving native speakers and that the psychometric testing followed and extended the protocol of the original validation study for the English version of FROM-16, allowing comparison of both studies. However, we have not formally assessed the measurement equivalence of the two language versions. Also, further research to assess differential item functioning is warranted.

We solely recruited partners and family members who provided informal care. This may be a limitation since informal caregiving may itself affect QoL. The main study limitation was the sample size with only 83 family members completing the questionnaire twice. Despite multiple recruiting pathways, the recruiting procedure was very laborious. This may hint that informal caregivers have neither the interest nor resources to engage in studies and may implicitly indicate the burden of caregiving itself. Consequently, selection bias cannot be ruled out.

Another limitation is that the exact test–retest interval is available for only 67 participants.

Further, we recognise that classical test theory (CTT) cannot address all aspects of the dimensionality of this measure. It will be necessary to retest the factor structure in a future study using item response theory (IRT).

Finally, there may be confusion between “family members” and “caregivers”, as family members may or may not be caregivers and vice versa. FROM-16 was developed and validated on subjects who were partners or family members of patients drawn from 26 medical specialties. These family members did not necessarily provide informal care, though may have done. FROM-16 was not designed to measure QoL of non-family health care providers [14]; whether it may be possible to use FROM-16 in this context should be the subject of future research.

## Conclusions

The two-domain structure of the original English version was not replicated for the German version. A bi-factor model with one general factor obtained better fit. Therefore, we recommend that results should be reported using the total score. As reliability and reproducibility, construct and criterion validity of the German FROM-16 can be considered as strong, we conclude that the German FROM-16 is a reliable and valid instrument to assess the impact of disease on partners and family members of patients in a generic context. More experience and data should be gathered in order to be able to reliably assess the transferability of the results on specific subgroups.

The FROM-16 fills a gap, as it measures QoL as an interdependent phenomenon between a family member and a patient; FROM-16 can now be used in a German-speaking context.

## Supplementary Information

**Additional file 1: Table S1. **Velicer’s MAP values showing average squared and average 4th-power partial correlations (Velicer et al., 2000).

## Data Availability

The datasets generated and/or analysed during the current study are not yet publicly available, but are available from the corresponding author on reasonable request.

## Abbreviations

CFA: Confirmatory Factor Analysis
CFI: Comparative fit index
DIF: Differential item functioning
EFA: Explanatory Factor Analysis
FRE: Flesch-Reading-Ease Score
FROM-16: Family Reported Outcome Measure-16 items
GHS: General Health Score
ICC: Intraclass Correlation Coefficient
TLI: Tucker-Lewis Index
PCA: Principal Component Analysis
RMSEA: Root mean square error of approximation
SRMR: Standardized root mean square residual
QoL: Quality of Life
WHOQOL-BREF: Abbreviated generic Quality of Life Scale developed through the World Health Organization
