# Supplementary material for: Validation of the German version of the Family Reported Outcome Measure (FROM-16) to assess the impact of disease on the partner or family member
Source: Health Qual Life Outcomes. 2021 Mar 24;19:106. doi: 10.1186/s12955-021-01738-4 (PMC7992821; doi:10.1186/s12955-021-01738-4)
Supplement: Supplementary file 1 — Additional file 1: Table S1. Velicer’s MAP values showing average squared and average 4th-power partial correlations (Velicer et al., 2000). [file 12955_2021_1738_MOESM1_ESM.docx]

**Supplementary table**

**Table S1 Velicer's MAP values showing average squared and average 4th-power partial correlations (Velicer et al., 2000)**

| Factors | 1 | 2 | 3 | 4 | 5 |
| --- | --- | --- | --- | --- | --- |
| MAP average squared partial r's | 0.097 | 0.026 | 0.023 | 0.037 | 0.042 |
| MAP4 based on the average 4th power of the partial r's | 0.015 | 0.002 | 0.002 | 0.004 | 0.005 |

Velicer, W. F., Eaton, C. A., & Fava, J. L. (2000). Construct explication through factor or component analysis: A review and evaluation of alternative procedures for determining the number of factors or components. In R. D. Goffin & E. Helmes (Eds.), Problems and solutions in human assessment: Honoring Douglas N. Jackson at seventy (pp. 41-71). New York, NY: Kluwer Academic/Plenum
